# Blood Metabolic Biomarkers of Occupational Stress in Healthcare Professionals: Discriminating Burnout Levels and the Impact of Night Shift Work

**DOI:** 10.3390/clockssleep7030036

**Published:** 2025-07-14

**Authors:** Andreea Petra Ungur, Andreea-Iulia Socaciu, Maria Barsan, Armand Gabriel Rajnoveanu, Razvan Ionut, Carmen Socaciu, Lucia Maria Procopciuc

**Affiliations:** 1 Department of Occupational Health, University of Medicine and Pharmacy “Iuliu Haţieganu”, Str. Victor Babes 8, 400347 Cluj-Napoca, Romania; andreea.ladaru@umfcluj.ro (A.P.U.); maria.opritoiu@umfcluj.ro (M.B.); armand.rajnoveanu@umfcluj.ro (A.G.R.); ionut.razvan@umfcluj.ro (R.I.); 2 Research Center for Applied Biotechnology and Molecular Therapy Biodiatech, SC Proplanta Str. Trifoiului 12G, 400478 Cluj-Napoca, Romania; csocaciu@proplanta.ro; 3 Department of Molecular Sciences, Medical Biochemistry, University of Medicine and Pharmacy “Iuliu Haţieganu, 400012 Cluj-Napoca, Romania; luciamariaprocopciuc@yahoo.com

**Keywords:** occupational burnout, night shift work, healthcare professionals, emotional exhaustion, low personal accomplishment, depersonalization, metabolomics, biomarker analysis, pathway analysis

## Abstract

Burnout syndrome is characterized mainly by three criteria (emotional exhaustion, depersonalization, and low personal accomplishment), and further exacerbated by night shift work, with profound implications for individual and societal well-being. The Maslach Burnout Inventory survey applied to 97 medical care professionals (with day and night work) revealed different scores for these criteria. Blood metabolic profiles were obtained by UHPLC-QTOF-ESI^+^-MS untargeted metabolomics and multivariate statistics using the Metaboanalyst 6.0 platform. The Partial Least Squares Discrimination scores and VIP values, Random Forest graphs, and Heatmaps, based on 99 identified metabolites, were complemented with Biomarker Analysis (AUC ranking) and Pathway Analysis of metabolic networks. The data obtained reflected the biochemical implications of night shift work and correlated with each criterion’s burnout scores. Four main metabolic pathways with important consequences in burnout were affected, namely lipid metabolism, especially steroid hormone synthesis and cortisol, the energetic mitochondrial metabolism involving acylated carnitines, fatty acids, and phospholipids as well polar metabolites’ metabolism, e.g., catecholamines (noradrenaline, acetyl serotonin), and some amino acids (tryptophan, tyrosine, aspartate, arginine, valine, lysine). These metabolic profiles suggest potential strategies for managing burnout levels in healthcare professionals, based on validated criteria, including night shift work management.

## 1. Introduction

Burnout syndrome is recognized as a significant global issue in occupational medicine, leading to widespread fatigue, job-related exhaustion, and dissatisfaction [1,2,3,4,5]. It is a state of stress characterized by mental exhaustion, physical fatigue, detachment from work, reduced competence, loss of energy, heightened irritability, sleep disturbances, and concentration difficulties. This condition can affect individuals across all professions and is often associated with depression and anxiety [6,7,8,9,10,11]. Burnout poses a substantial health concern for physicians and healthcare professionals, encompassing complex physiological and psychological dysfunctions caused by excessive stress. Recent reviews highlight it as a heterogeneous condition with considerable intra- and inter-individual variation, influenced by numerous risk factors [12,13,14,15].

Even though burnout is nowadays one of the most discussed mental health problems, it is not officially recognized as a mental disorder in most countries. Meanwhile, the number of publications related to burnout has increased considerably in the last decade, especially concerning causal agents, associated risk factors, and prevalence [14,15,16]. The variability in definitions and methods of measuring stress and burnout remains a challenge, as existing studies do not differentiate these syndromes based on endocrinological or clinically significant immunological changes [17]. Additionally, it is influenced by unavoidable risk factors such as age, gender, limited years of employment, and professional experience burnout as well as avoidable risk factors linked to emotional, organizational, environmental, and social elements [15,18].

The Maslach Burnout Inventory (MBI) [19,20] is a widely used questionnaire designed to assess burnout. It has various versions tailored to different target populations and evaluates three key dimensions of burnout [17,21,22]. These are depersonalization (DP), a detached or cynical attitude toward colleagues, emotional exhaustion (EE), the feelings of being overwhelmed by excessive workplace demands, lacking the emotional resources to cope with stress, and low personal accomplishment (PA), a perception of inefficiency and an inability to meet job demands effectively [19,23,24].

The risk factors for occupational burnout among medical personnel include night shifts, professional experience across multiple workplaces, job satisfaction, training, and work-related stress [14]. A systematic review categorized these risk factors into occupational and non-occupational factors [14,15,25,26], highlighting the significant impact of the COVID-19 pandemic, which exposed healthcare workers to a higher risk of infection [25,27,28,29]. Shift work is a common feature of medical training, the profession itself, and patient care. Studies have shown that sleep-deprived physicians working night shifts experience cognitive impairment particularly in hospital settings, making them more susceptible to burnout [30].

Numerous efforts have been made to distinguish burnout as either a psychological phenomenon or a clinical entity, aiming to clarify the biological connection between stress exposure and burnout, as well as to identify clinically applicable endocrine and immune biomarkers for burnout [17,18]. Exhaustion disorder is linked to metabolic abnormalities, fatigue, lack of energy, and a diminished capacity to mobilize energy, but the clinical burnout remain unclear, although over past decades, some potential biomarkers have been highlighted. These include metabolic indicators, such as cortisol, dehydroepiandrosterone sulfate (DHEAS), prolactin, natural killer (NK) cells, and C-reactive protein (CRP), which have been identified in case-control and cross-sectional cohort studies, along with disturbances in blood pressure [31]. The recent literature highlights the need for objective biomarkers linked to identifiable risks across different work environments to accurately diagnose burnout syndrome [11,17,31,32,33]. One study, using the Karasek questionnaire for 54 participants with burnout compared to 86 healthy controls examined factors (professional rank, sleep, job strain, social support, and anxiety and depression, suggesting that higher levels of glycemia, glycosylated hemoglobin, cholesterol, triglycerides, C-reactive protein, and thyroid-stimulating hormone, as well as lower levels of 25-hydroxyvitamin D, may serve as predictive markers of burnout. In contrast, models incorporating job strain, job satisfaction, anxiety, and insomnia did not effectively predict burnout [11]. Additionally, studies on thyroid hormones, prolactin, and cortisol have yielded inconclusive results, likely due to their diurnal and pulsatile nature [34,35,36]. Metabolomics is an emerging technology that serves as a powerful tool for identifying potential biomarkers and providing insights into their relevance as prospective biomarkers. Untargeted metabolomics is used to identify specific metabolite fingerprints in distinct sample groups, highlighting metabolites that differentiate the patient groups. High-throughput techniques such as gas chromatography or high-performance liquid chromatography coupled with mass spectrometry (LC-MS and LC-MS/MS) are commonly used. In addition, the identification and quantification of melatonin and N-acetyl serotonin in human plasma or urine [37], as well as other neurotransmitter biomarkers [38,39] are noted. Melatonin, a multifunctional regulator of circadian disruption, acts as a “chrono biotic” that adjusts and normalizes biological rhythms, coordinates critical processes and biomolecules (e.g., hormones and neurotransmitters), and regulates estradiol production [35,40].

In Romania, limited research or experimental studies have been conducted on burnout syndrome, its association with circadian dysfunctions, or exhaustion disorders, which negatively impact general health, especially mental well-being, workplace performance, and the attitude toward the occupational environment. In this context, we developed a retrospective study focusing on healthcare professionals with burnout syndrome, diagnosed based on the Maslach Burnout Inventory Questionnaire, with or without circadian disruption (e.g., night shift work). The study methodology included the use of this standardized questionnaire to assess burnout levels and select participants for blood sample collection. The samples were analyzed by an untargeted metabolomic approach using the UHPLC-QTOF-ESI^+^-MS technique. The goal was to identify potential metabolic biomarkers associated with burnout syndrome and their intensity levels.

## 2. Results

### 2.1. Untargeted Metabolomics to Discriminate the Metabolic Profiles of Night Work and Day Work Subjects

**Figure 1:**
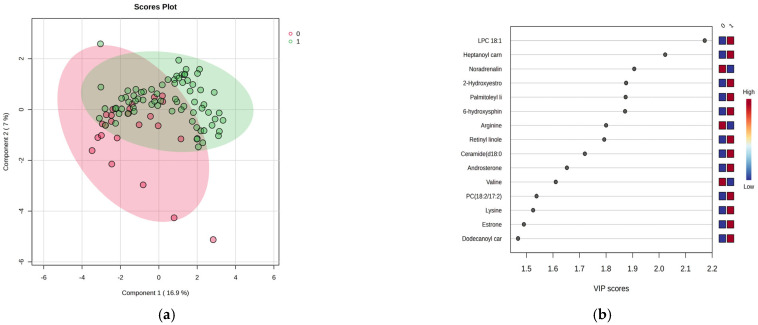

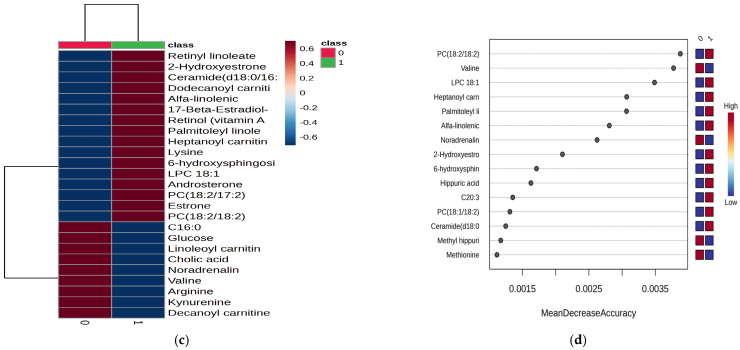
PLSDA score plot (**a**) and VIP score graph (**b**), the heatmap representation (**c**) and, RF-graph (**d**) for the 2 groups of subjects (day work-0 and night work-1).

Based on the data obtained through UHPLC-TOF-ESI^+^-MS analysis, 99 metabolites were identified and classified into five molecular categories, as detailed in Supplementary File S1. Using multivariate statistical methods, including Partial Least Squares Discriminant Analysis (PLSDA), Variable Importance in Projection (VIP) scores, Heatmap representation, and Random Forest (RF) analysis, the discrimination and significance of differences between the two subject groups were evaluated. Figure 1a–d presents the results of this analysis.

Figure 1a shows a moderate separation (23.9% variance explained) between night and day shift works, indicating distinct metabolic profiles. VIP scores above 1.9 and the Random Forest graph (Figure 1b,d) revealed significant serum increases for the top 15 metabolites, in conjunction with increased levels in the night shift group. The heatmap illustrates these variations. Of note were increases of lysine and decreases of arginine, methionine and valine, increases of lipid metabolites (fatty acids, hormones), and decreases in noradrenaline within the night work group. There were no significant increases in levels of PC18:2/18:2, heptanoyl carnitine, LPC 18:1, palmitoleyl linolenate, and linolenic acid.

### 2.2. Biomarker and Pathway Analysis of Metabolites for Group Night Work vs. Day Work

The Biomarker Analysis was applied to generate Receiver Operating Characteristic (ROC) curves, which assess the diagnostic ability of a biomarker, along with the Area Under the Curve (AUC) to determine the balance between sensitivity and specificity. Generally, biomarkers with the highest AUC values are considered the most promising for differentiating between the night work (group 1) and day work (group 0) groups. Table 1 presents the ranking of molecules with AUC values higher than 0.64 and *p*-values below 0.025, indicating significant differences between the groups. The log2FC values reflect the changes in biomarker levels in group 1 compared to group 0, with negative values indicating a downregulation and positive values indicating an upregulation.

**Figure 2:**
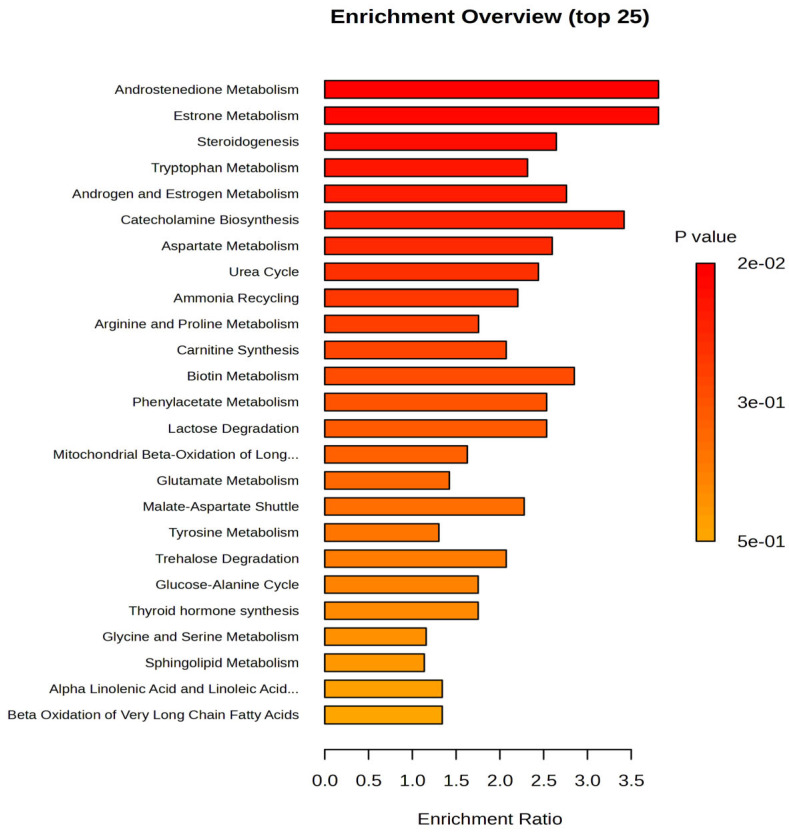
Metabolic pathways affected by night work compared to day work, as determined by Pathway Enrichment Analysis (enrichment ratios up to 3.5), considering all molecules selected for statistical analysis.

Based on these data, which provide additional insights, several metabolites were upregulated in the night work group, including 6-Hydroxysphingosine, Heptanoyl carnitine, LPC 18:1, palmitoleyl linolenate, retinyl linoleate, ceramide (d18:0/16:0), estrone, estradiol sulfate, and PC (18:2/18:2). On the other hand, downregulated levels were observed for noradrenaline, arginine, palmitoleic acid, glucose, and valine in the night work group. The cohort of 99 molecules was subjected to Pathway Enrichment Analysis based on *p*-values and enrichment scores, with a maximum score of 3.5, as shown in Figure 2.

Based on the enrichment ratios, the most affected pathways were androgen and estrogen metabolism (e.g., androstendione, estrone), steroidogenesis, followed by catecholamine biosynthesis (neurotransmitters derived from tyrosine, such as GABA and noradrenaline), and amino acid metabolism (involving tryptophan, aspartate, arginine, proline, and valine). Additionally, the pathways related to carnitine synthesis and acylation were also impacted.

### 2.3. Untargeted Metabolomics Analysis Considering the Burnout Criteria (DP vs. EE vs. PA)

**Figure 3:**
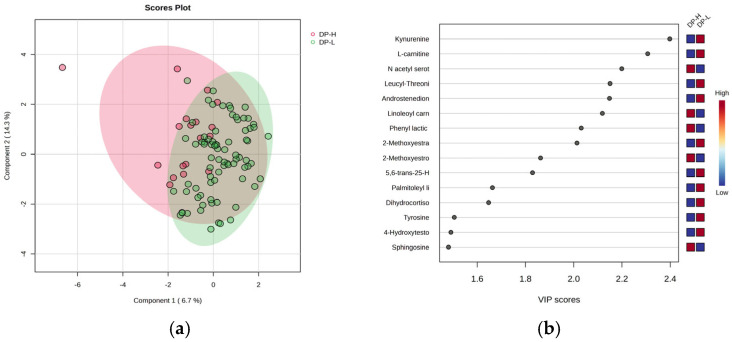
The PLSDA score plot (**a**) and VIP graph (**b**) showing the most significant molecules which differentiate subjects according to the DP criteria of burnout, with high (H) vs. low (L) levels.

**Figure 4:**
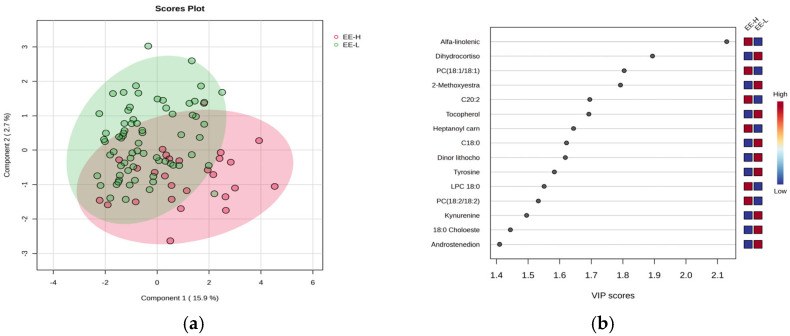
The PLSDA score plot (**a**) and the VIP graph (**b**) showing the most significant molecules which differentiate subjects with EE-H and EE-L burnout levels.

**Figure 5:**
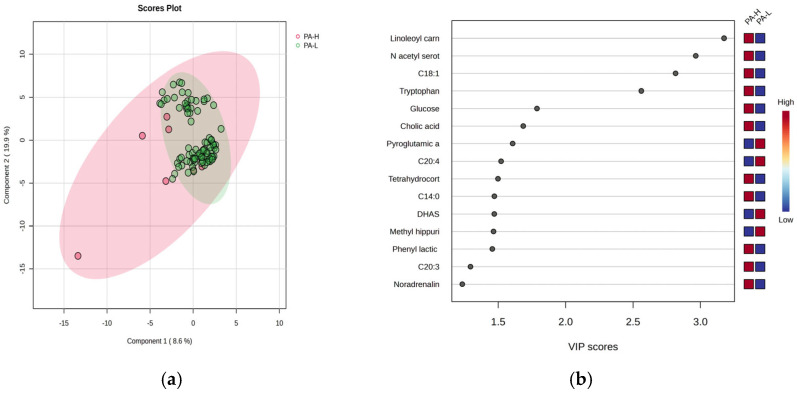
The PLSDA score plot (**a**) the VIP graph (**b**) showing the most significant molecules which differentiate subjects with PA-H vs. PA-L.

Considering the burnout criteria DP, EE, and PA for all subjects, the PLSDA graphs and VIP values are included in Figure 3, Figure 4, and Figure 5, respectively. The Supplementary File S3 includes the heatmaps (A) and RF-graphs (B) showing the most significant molecules which differentiate subjects according to the DP, EE, and PA criteria and low (L) versus high (H) levels of burnout. Figure 3 includes the PLSDA score plot (a) and VIP graph (b) showing the most significant molecules which differentiate subjects according to the DP criteria of burnout, with high (H) vs. low (L) levels.

The PLSDA score plot (Figure 3a) shows a covariance of 20%, with partial overlap between the DP-H and DP-L burnout groups. Group H exhibited a more homogeneous distribution compared to group L. The VIP scores (Figure 3b), with values above 2, revealed that kynurenine (a metabolite of tryptophan), L-carnitine, leucyl-threonine, androstenedione, and 17-beta methoxy estradiol had decreased levels in group H, while N-acetyl serotonin, methoxy estrone, and linoleoyl carnitine increased in group H. The Heatmap and RF analysis (Supplementary File S3) displayed a similar ranking, with additional decreases in gluconic acid and cholesterol ester C18:0.

Figure 4 includes the PLSDA score plot (a) and VIP graph (b), showing the most significant molecules which differentiate subjects according to the EE criteria of burnout, with high (H) vs. low (L) levels.

The PLSDA score plot (Figure 4a) shows also a moderate separation (18.6% variance explained) indicating a similar overlap between the burnout H and L groups. Fewer molecules had VIP scores above 2 (Figure 4b), including increased levels of alpha-linolenic acid and PC (18:1/18:1) in group H, while dihydrocortisol and methoxy estradiol decreased in group H. The Heatmap and RF analysis (Supplementary File S3) reveal a cohort of molecules with decreased levels in group H, including tocopherol, estrone, methoxy estradiol, stearic acid (C18:0), and cholesterol ester (C18:0).

Figure 5 illustrates the PLSDA score plot (a) and the VIP graph (b) with the most significant molecules which differentiate subjects with PA-H vs. PA-L.

In this case, the PLSDA score plot (Figure 5a) shows a covariance of 28.5%, with a similar overlap between group H (with few samples) and L group. Four molecules had VIP scores above 2 (Figure 5b), including increased levels of linoleoyl carnitine, oleic acid, N-acetyl serotonin, and tryptophan in group H. The Heatmap and RF analysis (Supplementary File S3) highlight a cohort of molecules with increased levels in group H, including cortisol, gluconic acid, retinol, methyl testosterone, and methoxy estradiol. As complementary information, the *t*-test graph (*p* < 0.5) based on multivariate analysis, is presented in Supplementary File S4, comparing the burnout criteria DP, EE, and PA for group 0 (day work—Figure S4A) and group 1 (night work—Figure S4B).

Additionally, a Debiased Sparse Partial Correlation (DSPC) network of molecular interactions within metabolic pathways was constructed using the KEGG pathway database. The DSPC network includes nodes representing input metabolites and edges denoting associations, with the top 20% of correlations ranked by *p*-value (Supplementary File S5).

This network, based on the relationships between different molecules within specific metabolic pathways, reveals that burnout primarily involves lipid metabolism with hormonal impacts (e.g., estrone, androstenedione, and cortisol derivatives), as well as lipophilic vitamins (retinol, tocopherol, ergocalciferol) and acylated carnitines, which are key molecules of lipid transport into mitochondria. Specific subnetworks for each class of metabolites are also provided in Supplementary File S5, established through Pathway Analysis for different levels of burnout, according to the criteria DP, EE, and PA.

### 2.4. Multivariate Statistics for Molecules Involved in Burnout Levels, Considering Only the Night Work Subjects

**Figure 6:**
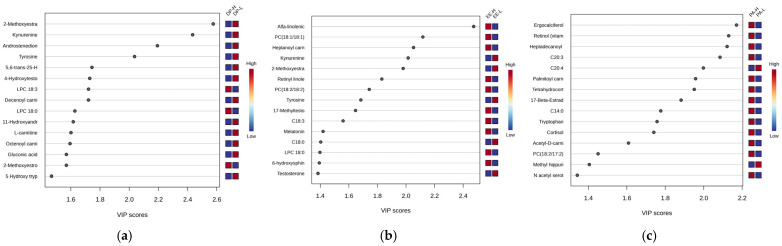
VIP scores including the top 15 molecules that may discriminate among night work subjects, considering the burnout criteria DP-H vs. DP-L (**a**), EE-H vs. EE-L (**b**), and PA-H vs. PA-L (**c**).

To specifically target the molecules from subjects with night work, the statistical analysis focused on the burnout criteria DP, EE, and PA for this subgroup. Figure 6 presents the most significant graphs (VIP scores and RF-graphs) highlighting the molecules responsible for discrimination between the three burnout criteria DP-H vs. DP-L, EE-H vs. EE-L, and PA-H vs. PA-L.

According to the VIP scores, the ranking of significant molecules differed in PA night work subjects compared to DP and EE groups. Most common molecules downregulated in high burnout were noticed when DP and EE criteria were considered. Noted were kynurenine, tyrosine, and steroid hormones (methoxyestradiol, androstenedione, hydroxytestosterone), acylated carnitines and C18-fatty acids, and phospholipids.

### 2.5. Specific Metabolic Profiles of Five Classes of Molecules

**Figure 7:**
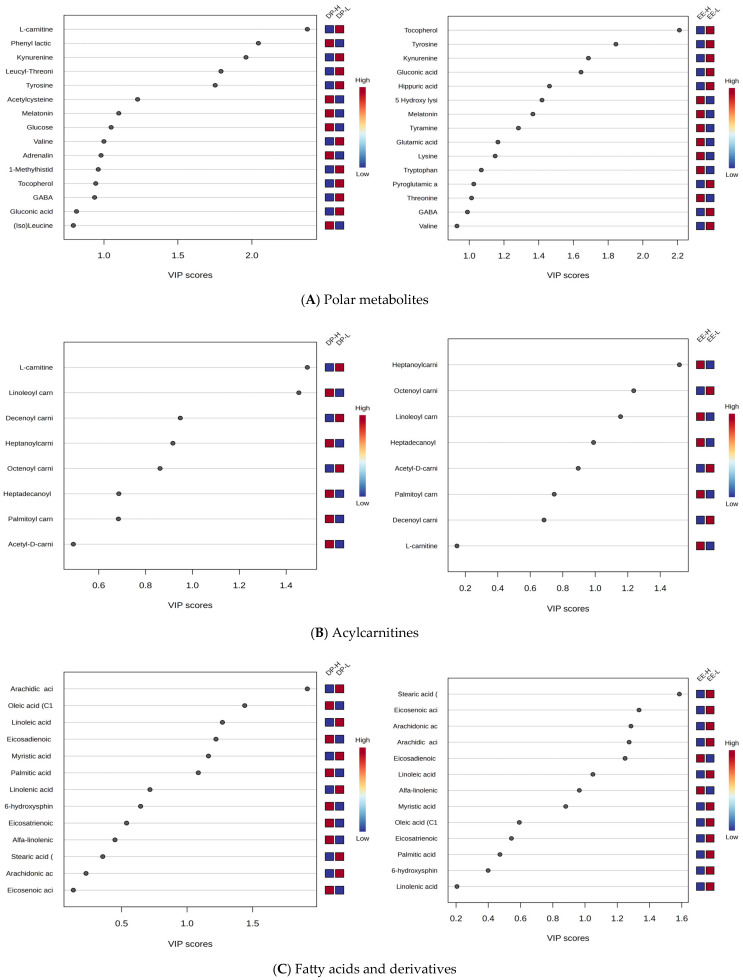

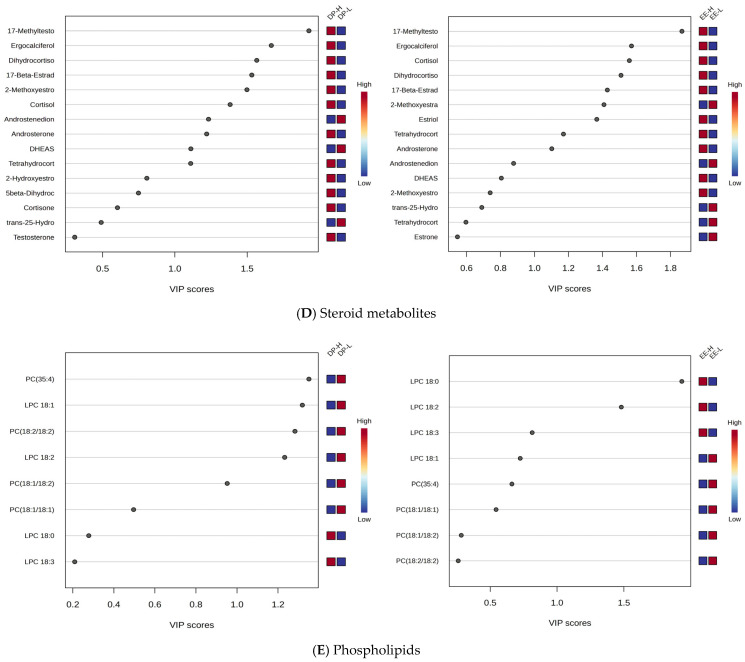
(**A**–**E**). VIP and RF graphs for each of the five (**A**–**E**) classes of metabolites, illustrating differences between groups DP-H vs. DP-L and EE-H vs. EE-L.

From the cohort of molecules identified (Supplementary File S1) and selected for statistical analysis, five classes of molecules were considered: polar metabolites, acylcarnitines, fatty acids and derivatives, steroid metabolites, and phospholipids, based on their involvement in specific metabolic pathways. For each class, the comparison was made according to criteria DP and EE, considered as most prominent criteria for burnout classification. Figure 7A–E illustrates comparatively the VIP scores to differentiate DP-H vs. DP-L and EE-H vs. EE-L.

Based on these results, specific variations across different classes of molecules were identified:

**Polar Metabolites**: Kynurenine, tyrosine, and tocopherol were significantly downregulated in both DP-H and EE-H groups, with VIP scores above 2.

**L-Carnitine and AcylCarnitines**: L-carnitine showed decreased levels, and acylated carnitines exhibited varying behaviors. Shorter-chain carnitines (C8-C10) had decreased levels in group H, while longer-chain carnitines (C16-C18) had increased levels in burnout groups H, for both DP and EE criteria.

**Free Fatty Acids**: These showed mostly decreased levels in both DP-H and EE-H subjects (e.g., stearic acid C18:0, linoleic acid C18:2, arachidic acid C20:0, arachidonic acid C20:4). However, C18 and C20-related fatty acids were more affected.

**Steroids**: Increased levels were observed for methyltestosterone, cortisol, dihydrocortisol, ergocalciferol, androsterone, estriol, and 17-beta estradiol in both DP and EE groups, while decreased levels were noted for dehydroepiandrosterone 3-sulfate (DHEAS), androstenedione, and estrone.

**Phospholipids**: Mainly C18-related phospholipids were affected (e.g., PC 18:1/18:1, 18:1/18:2, 18:2/18:2), with decreased levels in burnout H compared to L, while lysophospholipids (LPC 18:0, 18:2, 18:3) were increased in group H, especially for EE criteria.

These findings raised new questions regarding the behavior of metabolites specifically in subjects with night work only, prompting further investigation into this subgroup.

Table 2 summarizes the data and reflects the tendency of the most significant metabolites selected by multivariate analysis, considering highest VIP scores and Random Forest accuracy. The metabolic pathways mostly affected by both night shift and burnout levels are also highlighted.

The network analysis (Supplementary File S5) reveals two key molecular subnetworks, the lipid molecular network (including steroids, fatty acids, lipophilic vitamins, and acylated carnitines) and the network of polar molecules, mainly amino acid derivatives which are interconnected and predominantly affects the night shift, especially in high burnout classified by DP and EE criteria.

## 3. Discussion

Over the last decades, the relationship between work stress, burnout, and depression or anxiety have been intensely explored, as numerous studies in recent years focused on potential connections and overlaps [6]. Burnout-related hypotheses and investigations are mainly related to job demands following resource imbalances, as well as personal and organizational factors. Commonly, high job demands (workload or emotional stress) and insufficient job resources may be responsible for burnout onset, on one or all three dimensions of burnout: emotional exhaustion, depersonalization, and reduced personal accomplishment. 

Despite the significant impact of occupational burnout on healthcare quality, safety, and staff well-being, there are still challenges in defining and measuring burnout, largely due to the limited understanding of its pathophysiology [41].

In this context, the goal of the present study was to identify potential metabolic biomarkers which can be associated with burnout syndrome and their levels of severity. The investigation focused on burnout syndrome in 97 medical care professionals, applying the widely recognized MBI survey for measuring the different levels of burnout (high vs. low), according to the three criteria, emotional exhaustion (EE), depersonalization (DP), and personal accomplishment (PA). In parallel, the metabolic biomarkers correlated with burnout levels and different work environments (day work or circadian disruption due to night shift work) were evaluated. The methodology included an untargeted metabolomic approach using the UHPLC-QTOF-ESI^+^-MS technique, followed by a semi-targeted evaluation, focused on main metabolite classes influenced by the different levels of burnout.

Four objectives were considered: (1) comparing the metabolic profiles of day vs. night shiftwork independently on burnout criteria and levels, (2) correlations between the metabolic profile and burnout levels (high or low) considering DP, EE, and PA criteria (3) correlations between the metabolic profiles of night shift workers, considering DP, EE, and PA criteria, (4) correlation of the profiles of five metabolite classes affected by high vs. low burnout.

The main metabolic pathways influenced by night shift work affected steroid metabolism, including downregulation of Androstenedione, DHAS, in contrast with the increase in cortisol levels. Also, decreased levels of the main amino acids in parallel with an increase in lysine and acetyl serotonin levels, depletion of noradrenalin, a key neurotransmitter linked to chronic stress, were identified. Meanwhile, related to lipid metabolism, the upregulation of acylated carnitines (key molecules for the transport of acyl groups in mitochondria, for an increased beta-oxidation of lipid fuels), increased levels of several fatty acids, phospholipids, and lysophospholipids, especially the ones with C18 and C20 chains, were noticed.

When considering the three burnout criteria for the classification, it was found that the DP and EE criteria were more reliable and showed similarities regarding the metabolites responsible for high burnout levels. Therefore, when DP and EE criteria were considered for identifying putative biomarkers of burnout, the main findings showed similar behavior, as presented above. Meanwhile, the PA criteria gave less information; one explanation being that the less accurate classification of subjects with “low personal accomplishment” was from a smaller number of subjects being considered in the group PA-H in comparison to PA-L.

Our results are in strong alignment with the experimental literature data regarding the metabolites which may be related to burnout and thus, being better able to differentiate burnout from other anxiety-related disorders [42]. According to Humer et al. [43], in anxiety and depression, molecules involved in membrane formation and lipid metabolism, such as phospholipids, n-3 polyunsaturated fatty acids, sphingolipids, and DHAS, may be considered important biomarkers, along with those linked to carbohydrate metabolism. Higher levels of estrogens have been observed in women, while men showed lower levels of pregnenolone sulfate, PGF2, and adrenocorticotropic hormone. Association between burnout and immunological and endocrine alterations was also reported, e.g., reductions in blood levels of GABA, stearate, and 3-hydroxybutanoic acid as well as several hormones linked to high stress, including melatonin, cortisol, DHAS, ACTH, thyroid hormones, and prolactin [34,44,45,46,47,48]. A similar metabolomic study analyzed fasting and non-fasting plasma levels of 62 metabolites in 20 patients diagnosed with exhaustion disorder and compared with those from healthy controls. Using multivariate models, significant differences were observed among some amino acids and fatty acids, as well as sugar metabolites between the two groups, e.g., lysine and octadecenoic acid levels were increased in patients, while glutamine, glycine, serine, and gluconic acid levels were decreased [49]. The metabolic profile during sleep deprivation identified 27 blood metabolites (including tryptophan, serotonin, taurine, 8 acylcarnitines, 13 glycerophospholipids, and 3 sphingolipids) [50], higher levels of lysine, fatty acids (particularly oleic acid), and lower levels of glutamine, glycine, serine, and gluconic acid [40].

Mass spectrometry-based metabolomics studies on humans with sleep restriction have demonstrated changes in blood lipid metabolism (including sphingomyelins, phosphatidylcholines, lyso phosphatidylcholines, and acylated carnitines) as well as tryptophan metabolic pathways, including the involvement of serotonin, melatonin, and taurine. Changes in blood levels of hormones like cortisol and metabolites have also been valuable in assessing prolonged exposure to circadian disturbances [49,51]. A recent study using metabolomics is aligns closely with our data; certain phospholipid derivatives (phosphatidylserine C16:0/16:1 and phosphatidic acid C18:0) being identified as potential plasma biomarkers for major depressive disorders when compared to healthy subjects [52].

Tryptophan, a unique, scarce amino acid in cells, is crucial as a precursor for serotonin, N-acetyl serotonin, melatonin, and their derivatives, making its levels, along-side those of serotonin and melatonin, highly relevant in burnout [53]. Other authors reported melatonin and serotonin, which are activated by darkness and suppressed by light, may influence various physiological functions, and neurological status [32,35,37]. However, their pulsatile levels make it difficult to be correlated directly with the burnout levels. Our data did not show significant modifications of these molecules, but we noticed a downregulation of tryptophan and kynurenine (a metabolite of tryptophan), and upregulation of acetyl serotonin.

Our previous study recently published, analyzing urine samples from the same cohort of medical professionals [54], showed similar results: three main metabolic pathways and biomarkers identified as significant in diagnosing burnout being lipid metabolism, particularly involving steroid hormones (such as cortisol, cortisone, and androsterone metabolites); energetic metabolism, with long-chain acylated carnitines acting as transporters of free fatty acids to help regulate burnout levels; and catecholamine metabolism (neurotransmitters derived from tyrosine, including dopamine, adrenaline, and noradrenaline), as well as tryptophan metabolism (serotonin and melatonin metabolites, aspartate, arginine, and valine). Urine proved to be an effective and convenient biofluid for reflecting burnout levels and circadian disturbances.

To conclude, metabolomics based on UHPLC-QTOF-ESI^+^MS technology and multivariate statistics proved to be a useful tool to identify metabolic changes and potential biomarkers associated with burnout and night shift work among medical professionals.

Regarding our present study, there were some limitations: the sample size which was limited because of the number of available personnel from targeted hospitals and the number of female and male participants was un balanced, suggesting a higher prevalence of female personnel among medical professionals in Romania. Meanwhile, there were excluded potential co-factors and variables (smoking, alcohol, diet, and medication use). Further investigations are needed, on larger cohorts of professionals with burnout symptoms, in order to confirm the relevance of the metabolic biomarkers we have identified for diagnosis, including their role as predictors for the occupational burnout syndrome.

## 4. Materials and Methods

### 4.1. Study Design

This study adhered to the guidelines of the Declaration of Helsinki and the Conference for Coordination of Clinical Practice and was approved by the Ethics Committee for Scientific Research (DEP231/21.07.2023) at the “Iuliu Hațieganu” University of Medicine and Pharmacy Cluj-Napoca, Romania. Written informed consent was obtained from all participants. A total of 97 medical care professionals (doctors and nurses) were surveyed using the Maslach Burnout Inventory (MBI) in September–October 2023. Burnout levels for each subject were determined based on the three criteria: depersonalization (DP), emotional exhaustion (EE), and low personal accomplishment (PA) scores. The responses were collected through direct interviews, and participants were stratified into two groups: high burnout (high *n* = 6 and middle *n* = 15 scores) and low burnout (*n* = 76). The medical personnel were further classified based on their working conditions (night/day work) and according to the MBI questionnaire, with different scores for the three burnout criteria (DP, PA, and EE), as detailed in Table 3.

Additional information collected was through a thorough anamnesis, such as age, gender, and marital status, height, weight, blood pressure, lifestyle factors, and personal habits (smoking and alcohol consumption), as well as personal physiological data (for females, menstrual history), and the number of children. Additionally, we gathered information on previous diagnoses, medications, and vaccination status.

The occupational anamnesis covered aspects such as job type, field of activity, occupational history, work schedule, shifts, and length of service. Additional questions focused on night shifts, including their length and frequency, as well as sleep quality and quantity, with particular attention to symptoms associated with sleep deprivation.

As mentioned previously, burnout levels were assessed using the Maslach Burnout Inventory (MBI), which was licensed from Mind Garden. The inventory consists of 22 items: emotional exhaustion (EE) was evaluated through questions 1, 2, 3, 6, 8, 13, 14, 16, and 20; depersonalization (DP) was assessed through questions 5, 10, 11, 15, and 22; and personal accomplishment (PA) was evaluated through questions 4, 7, 9, 12, 17, 18, 19, and 21. Scoring was completed according to the MBI interpretation key.

### 4.2. Participants

Blood samples were collected from 97 healthcare professionals, including medical doctors and nurses, working in hospitals located in the Transylvania region of Romania. At the time of blood sampling, they were also provided with urine collection containers with instructions on how to collect the samples. Table 3 provides detailed information about participants, including the type of work (day shifts or night shifts) and gender distribution. Also, the table includes burnout evaluation according to burnout level scores, based on the MBI-HSS survey results. Middle and high scores were combined into a single category labeled as “high (H)”, as the number of high scores was significantly lower than that of middle scores. 

The inclusion criteria consisted of full and complete answers to all questionnaires, signed consent to participate in this study, consent for blood and urine sampling, active medical personnel (doctors, nurses, healthcare workers), at least 6 months of professional experience in healthcare, exposure to both day and night shifts for comparison, no known conditions that would interfere with the study (e.g., neurological or psychiatric disorders), and regular work schedules that involve burnout-related factors. The exclusion criteria included employees from sectors outside of healthcare, lack of informed consent, or refusal to provide biological samples.

### 4.3. Burnout Evaluation

As presented in Table 3, the participants’ burnout evaluation was measured according to the scores resulting from the MBI-HSS survey. Middle and high scores were combined into a single category labeled as “high (H)”, as the number of high scores was significantly lower than that of middle scores. The individual distribution of burnout scores were based on the criteria emotional exhaustion (EE), depersonalization (DP), and personal accomplishment (PA). The thresholds used to differentiate high (H) and low (L) scores were 17 for EE, 7 for DP, and 32 for PA. The Supplementary File S2 includes Graph (a) representing professionals working day shifts (*n* = 24), while Graph (b) represents those working night shifts (*n* = 73).

### 4.4. Methods Applied for Measurements

The blood samples were collected by venipuncture in sterile vacutainers without anticoagulant, in the morning before breakfast, on the same day of interview. The serum was kept at −80 °C until analysis and labelled using confidential numerical codes. All solvents and reagents used were of high purity. A volume of 0.8 mL mix of pure HPLC-grade Methanol and Acetonitrile (2:1 *v*/*v*) was added for each volume of 0.2 mL of serum. The mixture was vortexed to precipitate proteins, ultrasonicated 5 min and stored for 24 h at −20 °C to increase the protein precipitation. The supernatant was collected after the centrifugation at 12,500 rpm for 10 min (4 °C) and filtered through nylon filters (0.25 μm). Finally, it was placed in glass micro vials and introduced in the autosampler of the ultra-high-performance liquid chromatograph (UHPLC) before injection. Quality control (QC) samples were obtained by mixing 0.2 mL plasma from each sample at the beginning and after each 10 samples, for checking the reproducibility of LC-MS analysis.

The metabolomic profiling was performed by ultra-high-performance liquid chromatography coupled with electrospray ionization-quadrupole-time of flight-mass spectrometry (UHPLC-QTOF-ESI^+^-MS) using a Thermo Fisher Scientific, Waltham, MA, USA. UHPLC Ultimate 3000 instrument equipped with a quaternary pump, Dionex delivery system, and MS detection equipment with MaXis Impact (Bruker Daltonics, Bremen, Germany). The metabolites were separated on an Acclaim C18 column (5 μm, 2.1 × 100 mm, pore size of 30 nm) (Thermo Fisher Scientific, Waltham, MA, USA). The mobile phase consisted of 0.1% formic acid in water (A) and 0.1% formic acid in acetonitrile (B). The elution time was set for 20 min. The flow rate was set at 0.3 mL·min−1. The gradient for serum samples was 90–85% A (0–3 min), 85–50% A (3–6 min), 50–30% (6–8 min), 30–5% (8–12 min), and afterwards increased to 90% at min 20. The volume of injected extract was 5 µL and the column temperature was 25 °C. Doxorubicin hydrochloride (*m*/*z* = 581.3209) solution 0.5 mg/mL was added in parallel to QC samples as an internal standard. The MS ionization mode positive (ESI^+^) was applied, with a capillary voltage of 3500 V, the pressure for the nebulizing gas was 2.8 Barr, drying gas flow was 12 L/min, and drying temperature was 300 °C. The [M+1] (*m*/*z*) values were set between 100 and 800 Daltons. The control of the instrument and the data processing were conducted using the specific software TofControl 3.2, HyStar 3.2, Data Analysis 4.2 (Bruker, Daltonics, Bremen, Germany), and Chromeleon 7.3.1, respectively.

### 4.5. Statistical Analysis

Applying the UHPLC-QTOF-ESI^+^-MS analysis, a number of 1107 peaks were noticed. The acquired data were processed using the software Data Analysis 4.2. Initially, from the total ion chromatogram (TIC), specific algorithms were applied to generate the Base Peak Chromograms (BPC). The “Find Molecular Features” (FMF) algorithm was then used to create an advanced bucket matrix, which included details for each *m*/*z* value such as retention time, peak area, peak intensity, and the signal-to-noise (S/N) ratio.

For identification and statistical analysis, the following steps were taken: First, the chromatographic peaks with retention times under 0.8 min, intensities below 3000 units, S/N values below 10, and *m*/*z* values above 800 Daltons were removed. Next, *m*/*z* values were aligned using the online software (http://bioinformatica.isa.cnr.it/, accessed on 15 October 2024), retaining the common molecules found in more than 60% of the samples. The matrices for these metabolites, including their retention times, *m*/*z* values, and peak intensities, were statistically analyzed by converting them to a .csv file and importing them into the MetaboAnalyst 6.0 platform (https://www.metaboanalyst.ca/ (accessed on 30 March 2025)) for both multivariate and univariate analysis.

In total, 99 molecules were identified (Supplementary File S1) using the data provided from international databases such as the Human Metabolome Database (HMDB) and LipidMaps. The experimental *m*/*z* values were compared with the precursor ions [M+1] from these databases. The accuracy of the *m*/*z* values (theoretical minus experimental) was below 20 ppm.

The statistical analysis focused on untargeted multivariate analysis comparing group 1 (night shift) with group 0 (controls). Additionally, within each group (DP, EE, and PA), burnout low level (L) and burnout high level (H) were compared, as described earlier. Supervised discriminations between all groups were determined using Partial Least Squares Discriminant Analysis (PLSDA) and Random Forest (RF) prediction tests, illustrated by Heatmap clusters and correlations. The values of Variable Importance in Projection (VIP) and graphs of RF-Mean Decrease Accuracy (MDA) were calculated, providing a ranking of the most significant molecules for distinguishing between groups.

Biomarker Analysis was conducted using specific algorithms to generate ROC curves and calculate the Area Under the Curve (AUC), providing complementary predictions for potential biomarkers. Enrichment and Pathway Analysis were also performed based on the cohort of 99 identified molecules. Ultimately, the putative biomarkers for identifying metabolic disturbances induced by burnout were selected, based on both the untargeted analysis and the existing literature.

## 5. Conclusions

The burnout syndrome, characterized by emotional exhaustion, depersonalization, and low personal accomplishment, can be influenced by various stress factors, including night shift work in healthcare professionals. Through advanced metabolomics technologies, we identified four metabolic pathways and different classes of metabolites modified in patients with high burnout levels: the lipid metabolism, particularly in relation to steroid hormone synthesis (cortisol, androgens, and estrogens) (1), the amino acid metabolism (tryptophan, aspartate, arginine, tyrosine, valine, and lysine) and catecholamine metabolism, by downregulation of neurotransmitter noradrenaline (2), acylated carnitines, as vehicles of free fatty acids for mitochondrial energy (3), and upregulation of lipolysis with delivery of free fatty acids and increased synthesis of phospholipids, as a source of energy (4). These pathways offer potential strategies for managing burnout levels in healthcare professionals, considering also night shift work schedule management.

The use of a specific HPLC-QTOF-ESI^+^-MS protocols of metabolomics enabled accurate profiling of metabolites, as potential biomarkers with clinical relevance. Based on these findings, future prospective studies involving larger cohorts of patients could help refine the accuracy of biomarker diagnosis and enhance the prediction of occupational burnout syndrome including the metabolic stress in night shift workers at varying intensities, in more diverse cohorts. The clinical relevance of these findings should be applied at extended levels to implement future health policies.

## Figures and Tables

**Table 1:** The top 16 AUC values of potential biomarkers comparing the night work group (group 1) with the day work group (group 0). Log2FC values and sign indicate the intensity of a relative decrease (−) in group 1 (night work) vs. group 0 (day work).

Molecule	AUC	*p*	Log2FC	Molecule	AUC	*p*	Log2FC
6-Hydroxysphingosine	0.721	0.007	0.229	Retinol (vitamin A)	0.662	0.064	0.105
Heptanoyl carnitine	0.705	0.005	0.395	Estrone	0.658	0.028	0.351
LPC 18:1	0.703	0.002	0.514	Arginine	0.651	0.012	−0.340
Noradrenalin	0.695	0.004	−0.217	FA 16:1	0.651	0.006	−0.040
Palmitoleyl linolenate	0.684	0.008	0.796	2-Hydroxyestrone	0.650	0.008	0.490
Retinyl linoleate	0.678	0.015	0.375	Glucose	0.647	0.023	−0.112
Ceramide(d18:0/16:0)	0.674	0.019	0.357	Valine	0.644	0.017	−0.668
17-Beta-Estradiol-sulfate	0.667	0.023	0.073	PC (18:2/18:2)	0.642	0.018	0.256

**Table 2:** The most significant metabolites and metabolic pathways affected by night shift and burnout levels according to criteria DP, EE, and PA, where ⇓ represent decreased, ⇑ increased and NS nonsignificant changes in metabolites.

Metabolic Pathway	Molecules	Night vs. Day Work	Burnout (H vs. L)		
			DP-H vs. DP-L	EE-H vs. EE-L	PA-H vs. PA-L
Androgen and estrogen metabolism Steroidogenesis	Methoxy estradiol	⇓	⇓	⇓	⇓
	Estrone	⇑	NS	⇓	NS
	Hydroxy estrone	⇑	⇓	NS	NS
	Androstenedione, DHAS	⇓	⇓	⇓	NS
	Cortisol, Dihydrocortisol	⇑	⇑	⇑	NS
Polar metabolites (amino acid and derivatives) and	Trp, Kynurenine	⇓	⇓	⇓	NS
	Tyr, Tocopherol	⇓	⇓	⇓	NS
	Arg, Asp, Pro, Val	⇓	⇓	⇓	NS
	Lysine, Acetyl serotonin	⇑	⇑	NS	⇑
catecholamines	Noradrenalin	⇓	⇓	⇓	⇓
(Acyl)Carnitines	L-carnitine	⇓	⇓	⇓	NS
	Heptanoyl carnitine	⇑	⇑	⇑	NS
	Linoleyl carnitine	⇓	NS	NS	⇑
Free fatty acids	FA18:3	⇑	NS	⇑	NS
	FA 20:3	⇑	NS	NS	⇑
	FA 20:4	⇑	NS	NS	⇓
Phospholipids	PC (36:2, 36:4)	⇑	⇑	⇑	NS
	LPC (18:0, 18:1)	⇑	⇑	⇑	NS

**Table 3:** Demographic distribution, work schedule (day/night shifts), and burnout criteria scores (emotional exhaustion EE, depersonalization DP, low personal accomplishment PA) categorized as high (H) or low (L) based on MBI-HSS Survey thresholds for medical care professionals.

	Participants
Total	*n* = 97
Female/Male	86/11
Female (mean age ± SD)	46.4 ± 8.6
Male (mean age ± SD)	48.3 ± 10.2
Day/night work	24 day/73 night shift
EE-L (scores 0–16)	*n* = 72
EE-H (scores 17–51)	*n* = 25 including 17 night shift
DP-L (scores 0–6)	*n* = 79
DP-H (scores 7–18)	*n* = 18, including 13 night shift
PA-L (scores 32–48)	*n* = 90
PA-H (scores 0–31)	*n* = 7, including 3 night shift

## Data Availability

The original contributions presented in this study are included in the article/Supplementary Material. Further inquiries can be directed to the corresponding author.

## Supplementary Materials

The following supporting information can be downloaded at https://www.mdpi.com/article/10.3390/clockssleep7030036/s1.
