# Supplementary material for: Blood Metabolic Biomarkers of Occupational Stress in Healthcare Professionals: Discriminating Burnout Levels and the Impact of Night Shift Work
Source: Clocks Sleep. 2025 Jul 14;7(3):36. doi: 10.3390/clockssleep7030036 (PMC12285947; doi:10.3390/clockssleep7030036)
Supplement: Supplementary file 1 [file clockssleep-07-00036-s001.zip › Suppl file S1 identification .pdf]

Supplementary file S1. Serum molecules (n=99) separated using HPLC-QTOF-ESI^{+}-MS and identified, according to their m/z values.

N.B.The experimental m/z values (precursor ions [M+1]) were compared with the average of theoretical m/z values from the International database HMDB (Human Metabolomic DataBase). The accuracy of (theoretical – experimental) m/z values was below 20 ppm. The ID HMDB codes are mentioned in column 3.

[M+1] precursor ion	Identification	ID (HMDB)
	**Polar metabolites (n=35)**	
105.0348	Gama-aminobutyric acid (GABA)	HMDB0000112
119.0869	Diamino butyric acid (DABA)	HMDB0006284
130.0658	Pyroglutamic acid	HMDB0304793
151.1152	Phenyl lactic acid	HMDB0000814
181.9543	Glucose	HMDB0000122
197.1196	Gluconic acid	HMDB0000625
116.0727	L-Proline	HMDB0000162
118.0898	Valine	HMDB0000883
120.0832	Threonine	HMDB0000167
132.0801	(Iso)Leucine	HMDB0000172
133.0348	Asparagine	HMDB0000168
134.0615	Aspartic acid	HMDB0000191
138.0927	Tyramine	HMDB0000306
147.0824	Lysine	HMDB0000182
148.0635	Glutamic acid	HMDB0000148
150.0614	Methionine	HMDB0000696
162.0574	L-carnitine	HMDB0000062
163.1175	5 Hydroxy lysine	HMDB0000450
164.0404	Hydroxy glutamic acid	HMDB0001344
170.0669	Noradrenalin	HMDB0015451
175.1341	Arginine	HMDB0000517
177.1672	Serotonin	HMDB0000259
180.1390	Hippuric acid	HMDB0000714
182.1584	Tyrosine	HMDB0000158
184.1005	Adrenalin	HMDB0000068
188.0049	N-acetyl spermidine	HMDB0001276
194.1288	Methyl hippuric acid	HMDB00859
205.1980	Tryptophan	HMDB0000929
209.1580	Kynurenine	HMDB0000684
219.1797	N-acetyl serotonin	HMDB0001238
221.1214	5 Hydroxy tryptophan	HMDB0000471
233.1580	Leucyl-Threonine	HMDB0028939
233.2310	Melatonin	HMDB0001389
394.3654	N acetyl serotonin glucuronide	HMDB60833
417.2716	Tocopherol	LMPR02020001
	**Fatty acids, and derivatives (n=22)**	
229.146	Myristic acid C14:0	HMDB0000806
255.0007	Palmitoleic acid (C16:1)	HMDB60082
257.2524	Palmitic acid (C16:0)	HMDB0000220
279.1657	Alfa-linolenic acid	HMDB0001388
279.2384	Linolenic acid (C18:3)	HMDB0003073

281.2544	Linoleic acid (C18:2)	HMDB0000673
283.2309	Oleic acid (C18:1)	HMDB0000207
285.2967	Stearic acid (C18:0)	HMDB0000827
287.2403	Retinol	HMDB0000305
298.3541	(d18:2) sphingosine	LMSP01080011
300.2144	(d18:1) Sphingosine	HMDB0000252
305.2726	Arachidonic acid (C20:4)	HMDB0001043
307.2551	Eicosatrienoic acid (C20:3)	HMDB0002925
309.2360	Eicosadienoic acid (C20:2)	HMDB0005060
311.1926	Eicosenoic acid (C20:1)	HMDB0002231
313.2426	Arachidic acid (C20:0)	HMDB0002212
316.3296	6-hydroxysphingosine	LMSP01080003
355.2905	Prostaglandin F2	HMDB0001139
501.1286	Palmitoleyl linolenate	LMFA07010121
505.3556	Linoleyl palmitate	LMFA07010126
540.4376	Ceramide(d18:0/16:0)	HMDB0011760
547.2044	all-trans-retinyl linoleate	LMPR01090016
**AcylCarnitines (n=8)**		
204.1273	Acetyl-D-carnitine	HMDB0240771
274.2804	Heptanoylcarnitine (C7:0)	HMDB0013238
286.3174	Octenoyl carnitine (C8:1)	HMDB0013324
314.2406	Decenoyl carnitine (C10:1)	HMDB0241072
344.2168	Dodecanoyl carnitine (C12:0)	HMDB0000944
400.3913	Palmitoyl carnitine (C16:0)	HMDB0240952
414.3365	Heptadecanoyl carnitine (C17:0)	HMDB0006210
424.3764	Linoleoyl carnitine (C18:2)	HMDB0006681
**Steroids and vitamins (n=25)**		
271.2698	Estrone	HMDB0000145
277.1839	19-norandrosterone	HMDB0002697
287.1727	2-Hydroxyestrone	HMDB0000343
287.1864	Androstenedione	HMDB0000053
289.1413	Estriol	HMDB0000151
289.1617	Testosterone	HMDB0000234
291.2598	Androsterone	HMDB0000031
301.1493	2-Methoxyestrone	HMDB0000010
303.2394	2-Methoxyestradiol-17beta	HMDB0000405
303.2606	17-Methyltestosterone	HMDB0015655
305.1787	4-Hydroxytestosterone	HMDB0246468
307.2203	11-Hydroxyandrosterone	HMDB0002984
317.2187	Pregnenolone	HMDB0000253
349.2733	5beta-Dihydrocorticosterone	LMST02030280
361.2349	Cortisone	HMDB0002802
363.2248	Cortisol	HMDB0000063
365.1479	Tetrahydrocortisone	HMDB0000903
365.2885	Dihydrocortisol	HMDB0003259
367.2282	Tetrahydrocortisol	HMDB0000949
369.2536	Dehydroepiandrosterone 3-sulfate (DHEAS)	HMDB0001032
397.3046	Ergocalciferol (Vit D2)	HMDB0000161
401.2792	trans-25-Hydroxyvitamin D3	HMDB0006721

409.2682	Cholic acid	HMDB0000619
433.2723	17-Beta-Estradiol-sulfate	HMDB0041620
653.6169	18:0 Cholesterol ester	HMDB0010368
**Phospholipids (n=9)**		
518.3183	LPC 18:3	HMDB0010387
520.3557	LPC 18:2	HMDB0010386
522.3662	LPC 18:1	HMDB0002815
524.3830	LPC 18:0	HMDB0010384
539.3818	LPE 22:0	HMDB11520
768.6063	PC(35:4)	LMGP01010931
782.6044	PC(18:2/18:2)	HMDB0008138
784.5803	PC(18:1/18:2)	HMDB0008072
786.6206	PC(18:1/18:1)	HMDB0000593
