# Supplementary material for: Blood Metabolic Biomarkers of Occupational Stress in Healthcare Professionals: Discriminating Burnout Levels and the Impact of Night Shift Work
Source: Clocks Sleep. 2025 Jul 14;7(3):36. doi: 10.3390/clockssleep7030036 (PMC12285947; doi:10.3390/clockssleep7030036)
Supplement: Supplementary file 1 [file clockssleep-07-00036-s001.zip › Suppl file S2.pdf]

Supplementary file S2. Individual scores for Depersonalization (DP), Emotional Exhaustion (EE), and Personal Accomplishment (PA) Classified by Day Work (a) and Night Work (b).

Based on the data presented in Table 1 the night work group (b) shows positive correlations between higher score levels and increased burnout for the EE and DP criteria, with mean values of 23.35 and 8.53, compared to 11.19 and 2.95, respectively, in the day work group. Additionally, this trend is evident in a larger number of subjects, such as participants 4, 8, 55, 61, and 69.

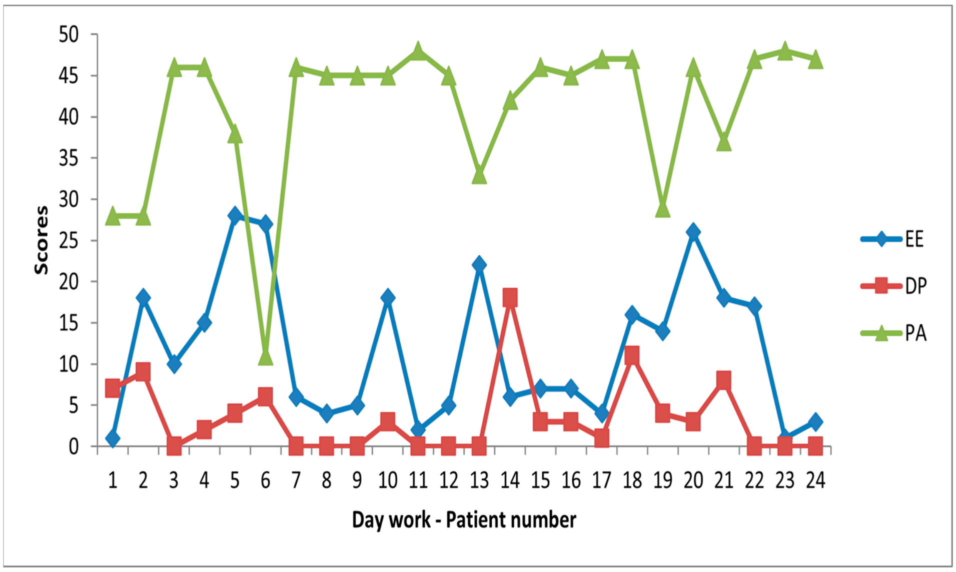

(a)

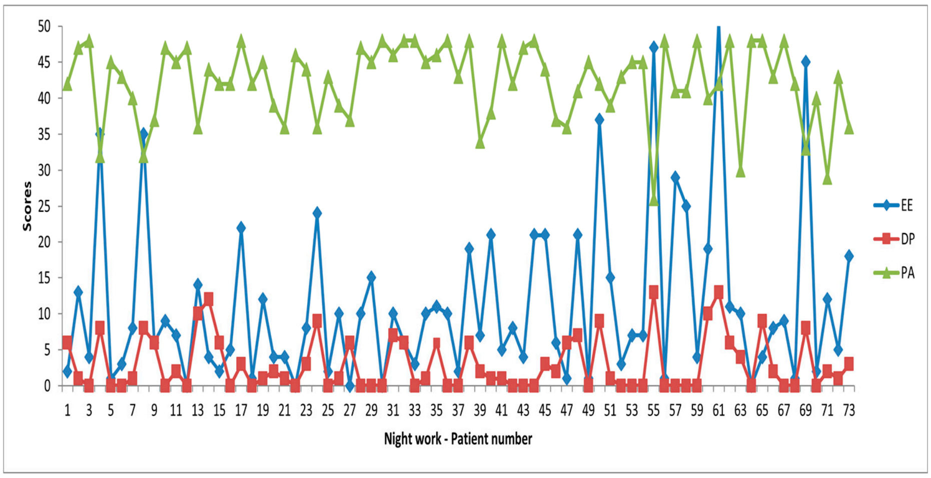

(b)
