# Supplementary material for: Blood Metabolic Biomarkers of Occupational Stress in Healthcare Professionals: Discriminating Burnout Levels and the Impact of Night Shift Work
Source: Clocks Sleep. 2025 Jul 14;7(3):36. doi: 10.3390/clockssleep7030036 (PMC12285947; doi:10.3390/clockssleep7030036)
Supplement: Supplementary file 1 [file clockssleep-07-00036-s001.zip › Suppl file S3.pdf]

Supplementary file S3. Heatmaps (A) and RF-graphs (B) showing the most significant molecules which differentiate subjects according to the DP, EE and PA criteria, between High (H) and low(L) levels of burnout.

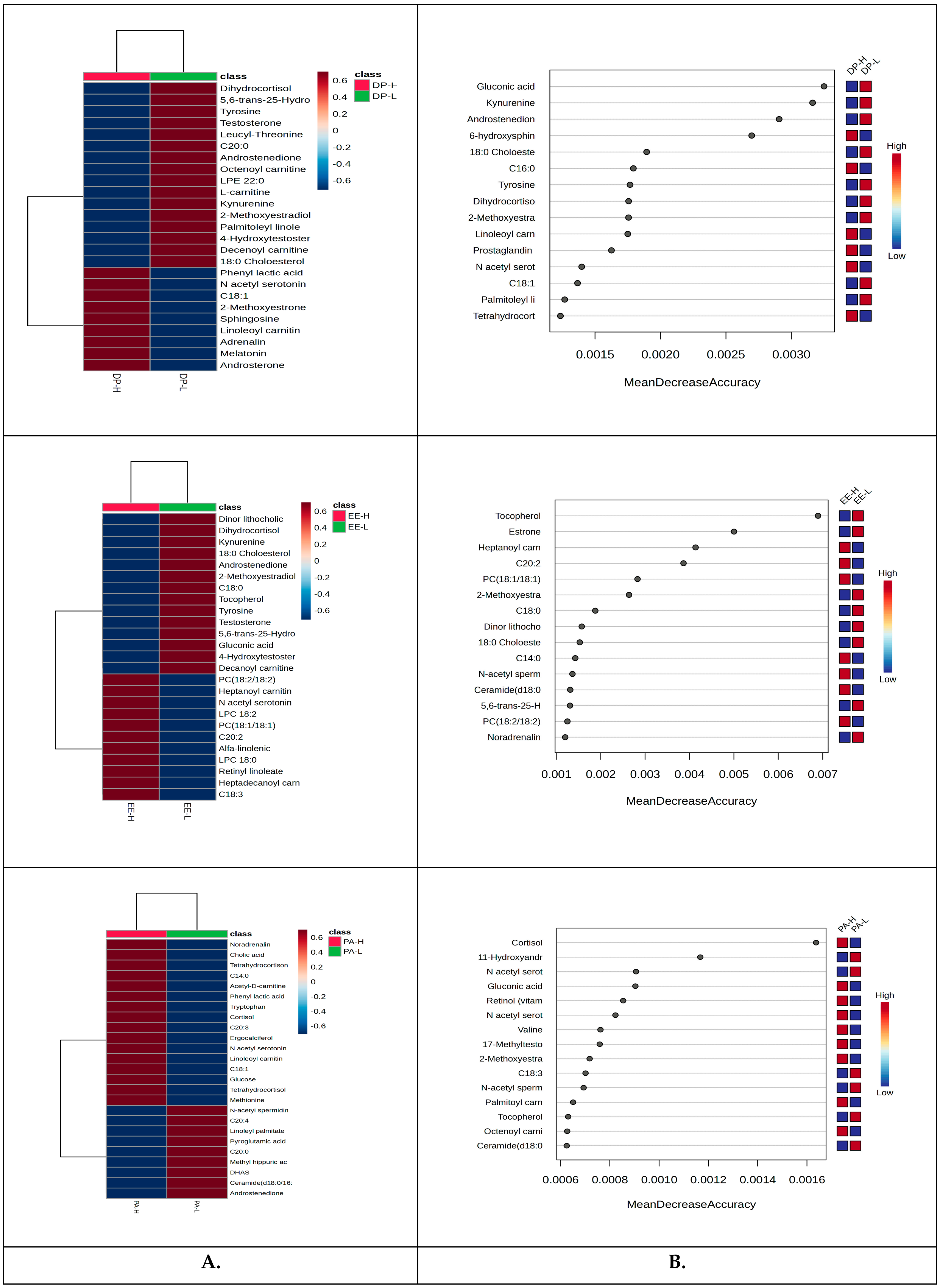

**A.**	**B.**
