# Supplementary material for: Blood Metabolic Biomarkers of Occupational Stress in Healthcare Professionals: Discriminating Burnout Levels and the Impact of Night Shift Work
Source: Clocks Sleep. 2025 Jul 14;7(3):36. doi: 10.3390/clockssleep7030036 (PMC12285947; doi:10.3390/clockssleep7030036)
Supplement: Supplementary file 1 [file clockssleep-07-00036-s001.zip › Suppl file S4 T test.pdf]

Supplementary file S4. The representation of the T-test according to the multivariate analysis for the discrimination considering the burnout criteria DP, EE, PA for groups 0 (day work- Figure S4A) and group 1 (night work-Figure S4B)

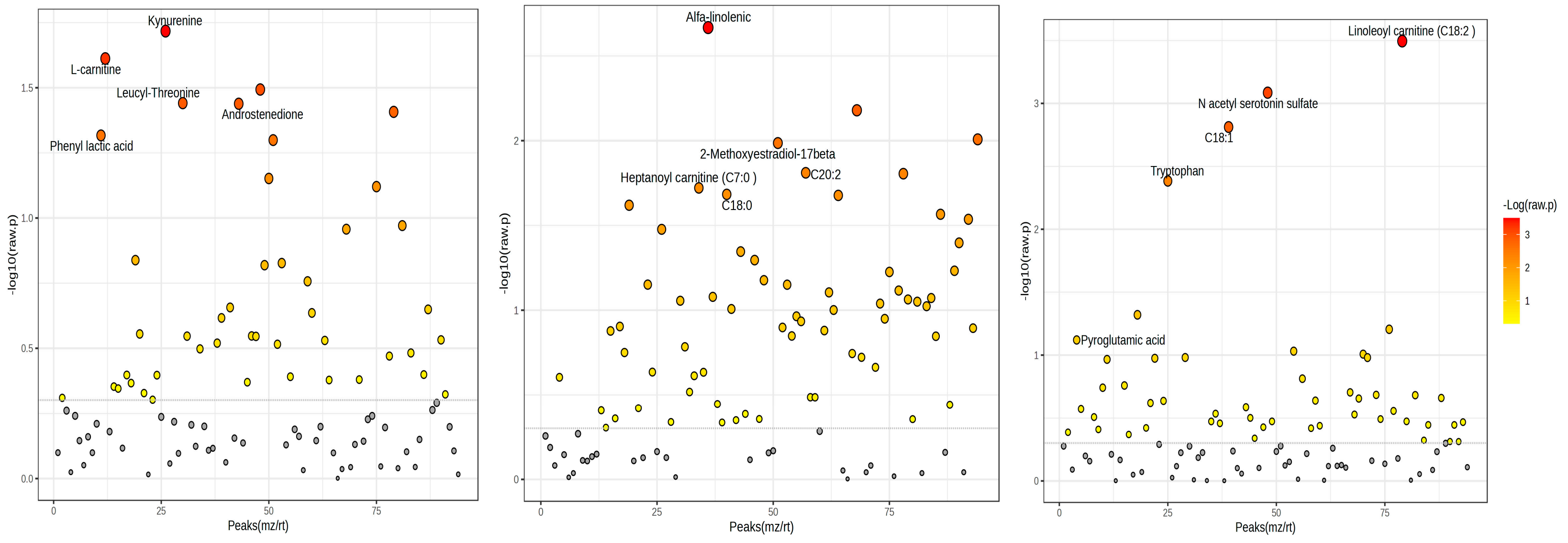

(a) (b) (c)

Figure S4A. The representation of the T-test according to the multivatriate analysis for the discrimination of groups 0 and 1, considering the criteria DP(a), EE (b), PA (c)

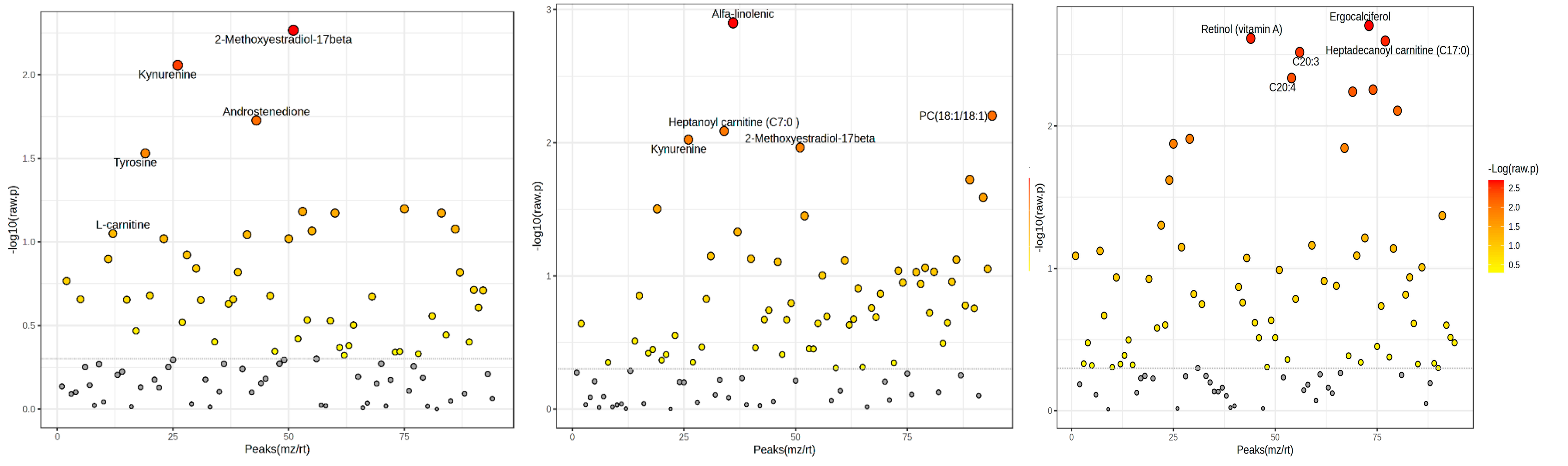

(a) (b) (c)

Figure S4B. The representation of the T-test according to the multivariate analysis for the night work group, considering the criteria DP(a), EE (b), PA (c)DP(a), EE (b), PA (c)
