# Supplementary material for: Blood Metabolic Biomarkers of Occupational Stress in Healthcare Professionals: Discriminating Burnout Levels and the Impact of Night Shift Work
Source: Clocks Sleep. 2025 Jul 14;7(3):36. doi: 10.3390/clockssleep7030036 (PMC12285947; doi:10.3390/clockssleep7030036)

Supplementary file S5. DSPC networks and subnetworks of different classes of molecules, identified by Pathway Analysis for night shift subjects, considering criteria DP, EE and PA.

Lipid molecular network and steroid subnetowork

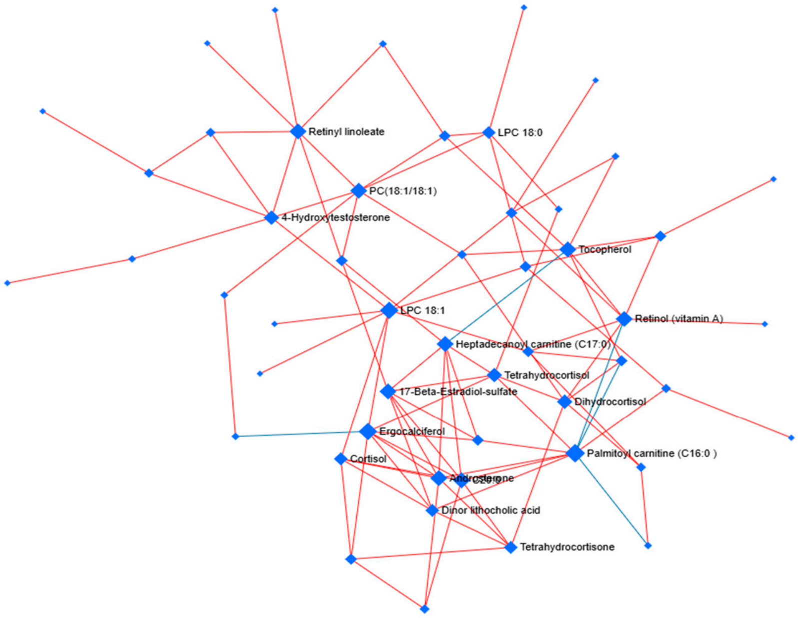

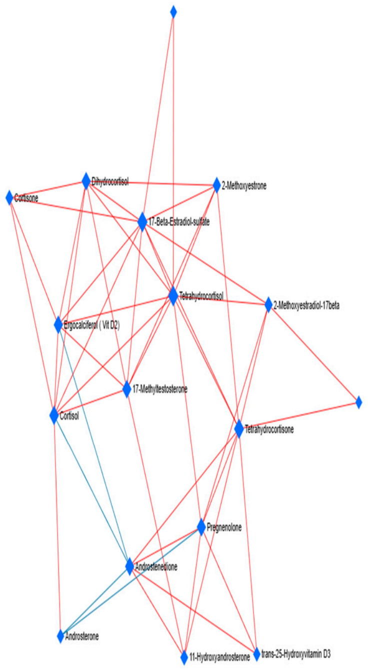

Amino acid network

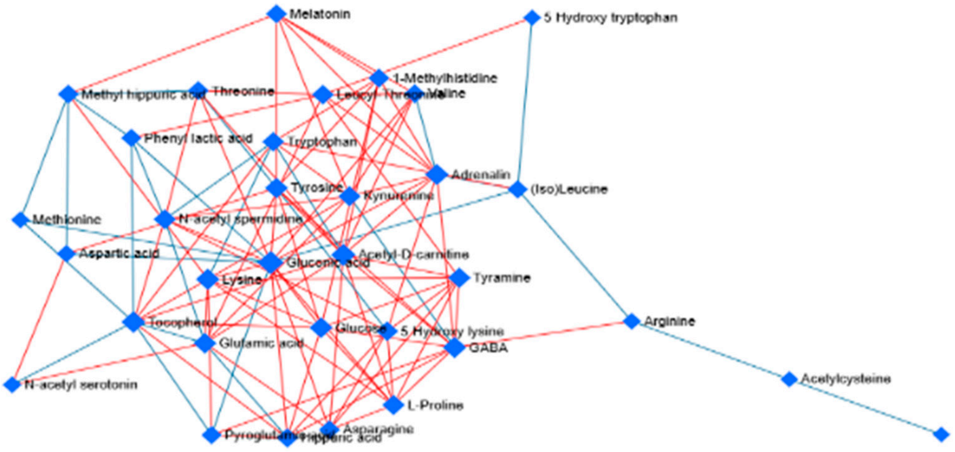

Supplement: Supplementary file 1 [file clockssleep-07-00036-s001.zip › Suppl file S5 Network.pdf]
